# Investigation of the Oxidation Behaviour of Ti and Al in Inconel 718 Superalloy During Electroslag Remelting

**DOI:** 10.1038/s41598-018-23556-3

**Published:** 2018-03-27

**Authors:** Shengchao Duan, Xiao Shi, Mingtao Mao, Wensheng Yang, Shaowei Han, Hanjie Guo, Jing Guo

**Affiliations:** 1 School of Metallurgical and Ecological Engineering, University of Science and Technology Beijing (USTB), Beijing, 100083 P. R. China; 2 Beijing Key Laboratory of Special Melting and Preparation of High-End Metal Materials, Beijing, 100083 P. R. China; 3 Research Institute of High Temperature Materials, Central Iron and Steel Research Institute (CISRI), Beijing, 100081 P. R. China

## Abstract

In the current study, the thermodynamics of the slag-metal equilibrium reaction between Inconel 718 Ni-based alloy and CaF_2_-CaO-Al_2_O_3_-MgO-TiO_2_ electroslag remelting (ESR)-type slags were systematically investigated in the temperature range from 1773 to 1973 K (1500 to 1700 °C). The equilibrium Al content increased with increasing temperature, whereas the equilibrium Ti content decreased with increasing temperature at a fixed slag composition. The important factors for controlling the oxidation of Al and Ti in the Inconel 718 superalloy were TiO_2_ > Al_2_O_3_ > CaO > CaF_2_ > MgO in ESR-type slag and Al > Ti in a consumable electrode. The conventional method of sampling by means of a quartz tube could result in contamination of the molten metal and changes in the size of the “special reaction interface”. Therefore, a novel method was used in the present study to investigate the slag-metal reaction kinetics to accurately obtain the kinetic parameters. The mass transfer coefficient was determined by coupling with the kinetic model derived from the assumption that the reaction rate ([Al] + (TiO_2_) = [Ti] + (Al_2_O_3_)) was controlled by the mass transfer of [Ti], [Al], (TiO_2_) and (Al_2_O_3_) in the boundary layer, respectively.

## Introduction

**Figure 1:**
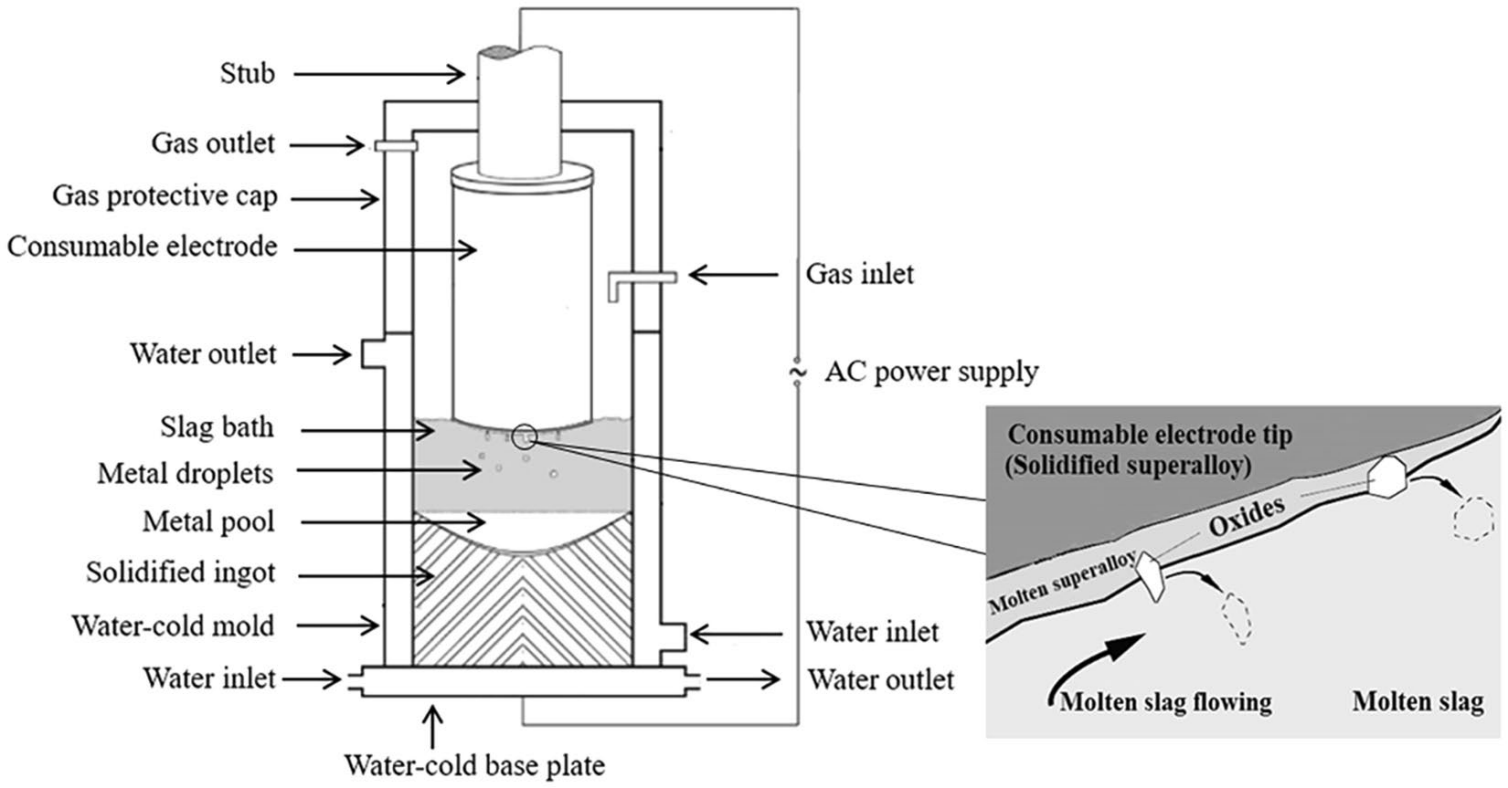
Schematic of the electroslag remelting (ESR) setup.

Inconel 718 is extensively used for aerospace and other components that operate at high temperatures and in corrosive environments, where materials with both high strength and excellent corrosion resistance are required^1–5^. The increasing demand for alloys with remarkable comprehensive performance necessitates the development of alloys with highly uniform microstructures and highly homogeneous composition. Electroslag remelting (ESR) is well known for cleanliness and homogeneity of the solidification structure of the resultant ingot. The poor homogeneity of the ingot’s composition, however, illustrates that some problems with the process remain unsolved^6,7^. Strong chemical reactions usually occur at the electrode-slag interface (ESI) or the droplet-slag interface (DSI) as well as at the metal pool-slag interface (MSI), as shown in Fig. 1^8^. Consequently, in some cases, the concentrations of critical elements cannot be maintained within specifications or the elements cannot be uniformly distributed along the height of the ingots during the ESR process^9^.

Numerous studies of the aforementioned issues have been reported. The approaches mainly involve encapsulating a preformed consumable electrode by covering the cap between the electrode and water-cooled mold and blowing inert gases onto the surface of the slag bath^10–12^, adding an aluminium shot to the slag bath or an aluminium coating to the electrodes to reduce oxidation of the slag caused by rust on the surface of the consumable electrode and by air above the slag pool^12–14^. Furthermore, the oxidation of Ti and Al in Inconel 718 is unlikely to be avoided, as demonstrated by the following equilibrium between Al_2_O_3_ and TiO_2_ in the CaF_2_-CaO-Al_2_O_3_-based slag^6^:

1 $$\begin{array}{c}4[{\rm{Al}}]+3({{\rm{TiO}}}_{2})=3[{\rm{Ti}}]+2({{\rm{Al}}}_{2}{{\rm{O}}}_{3})\\ {\rm{lg}}\,K=\,{\rm{lg}}\,\frac{{a}_{ \% ,{\rm{Ti}}}^{3}{a}_{{\rm{R}},{{\rm{Al}}}_{2}{{\rm{O}}}_{3}}^{2}}{{a}_{ \% ,{\rm{Al}}}^{4}{a}_{{\rm{R}},{{\rm{iO}}}_{2}}^{3}}=\frac{35300}{T}-9.94\end{array}$$

Therefore, determining the thermodynamic equilibrium between the reactive elements considered and oxide components in ESR slag is very important to diminish the loss of Al and Ti during the ESR process.

In the present study, the dependence of the equilibrium content of Al in Inconel 718 on each component in CaF_2_-CaO-Al_2_O_3_-MgO-TiO_2_ slag in the temperature range from 1773 to 1973 K (1500 to 1700 °C) was investigated. Meanwhile, the effect of the Al content on the equilibrium Ti content in the Ni-based alloy was also studied, through which the appropriate composition of the consumable electrode was obtained.

Under conventional conditions, the melt-quenching method is used to investigate chemical reaction kinetics; that is, slag and metal samples are periodically collected using quartz tubes and quenched in ice water, which is the most commonly used approach^15^. However, the liquid metal containing reactive elements such as Al, Ti, rare-earth elements (REE), *etc*., can react with the quartz tube, viz., 4[Al] + 3SiO_2_ = 2Al_2_O_3_ + [Si], resulting in contamination of the molten metal^16^. In addition, the net flux of species *i* in the slag or the metal phase can be written as

2 $$\frac{{\rm{d}}{C}_{i}}{{\rm{d}}t}={k}_{i}\frac{A}{V}({C}_{i}^{\ast }-{C}_{i})$$

where *k*_*i*_, $\frac{A}{V}$, ${C}_{i}^{\ast }$ and *C*_*i*_ represent the mass transfer coefficient of component *i* (m/s), the ratio of the interfacial reaction area to the effective liquid melt volume (we introduce the concept of “specific reaction interface” (SRI) (1 /m) in the present study, and concentrations at the slag-metal interface and in the bulk (mol/m^3^), respectively. The preceding experimental technique can increase the SRI value, either because of the turbulence of the reaction interface or because of a decrease in the absolute amount of melts during sampling^17^. Consequently, the changes in the value of the SRI cause the integration of Eq. (2) to become more complex and inconvenient^16^.

To solve the aforementioned problems, we investigated the reaction kinetics of Inconel 718 alloy with CaF_2_-CaO-Al_2_O_3_-MgO-TiO_2_ slags using laboratory-scale experiments, which we conducted under the same experimental conditions (concentration, temperature, and protective atmosphere) by using several identical crucibles with the same melt contents. Meanwhile, we derived a kinetic model using the fundamental equation of heterogeneous reaction kinetics based on the concept of effective boundary layer proposed by Wagner to obtain kinetic parameters^18^.

## Thermodynamic considerations

According to reaction (1), the relationship between the equilibrium content of Al in Inconel 718 and variables such as the activity *a*_*i*_ of components *i* in CaF_2_-CaO-Al_2_O_3_-MgO-TiO_2_ slags, chemical constitution of the consumable electrode, and the temperature of the slag can be obtained by simple mathematical derivation.

3 $$\mathrm{lg}[ \% \,{\rm{Al}}]=\frac{1}{4}\{{\rm{lg}}\,\frac{{a}_{{\rm{R}},{{\rm{Al}}}_{2}{{\rm{O}}}_{3}}^{2}}{{a}_{{\rm{R}},{{\rm{TiO}}}_{2}}^{3}}-4\,{\rm{lg}}\,{f}_{ \% ,{\rm{Al}}}+3\,{\rm{lg}}\,{f}_{ \% ,{\rm{Ti}}}+3\,\mathrm{lg}[ \% \,{\rm{Ti}}]-(\frac{35300}{T}-9.94)\}$$

Calculating the equilibrium content of Al requires that the relevant parameters be obtained: (I) The mass action concentration (activity) *N*_*i*_ of each component in the slag can be calculated using the reported activity model (for details of the modelling and solution procedure, see Eqs (1) through (3) in ref.^19^) based on the ion and molecule coexistence theory (IMCT)^20^. The basic meaning of *N*_*i*_ is consistent with the traditionally applied activity *a*_*i*_ in the slag, in which pure solid matter is chosen as the standard state and mole fraction is selected as a concentration unit. According to the hypothesis of the IMCT^20–22^, CaF_2_-CaO-Al_2_O_3_-MgO-TiO_2_ slag is composed of four simple ions (Ca^2+^, Mg^2+^, O^2−^ and F^−^), two simple molecules (Al_2_O_3_ and TiO_2_), and approximately 15 complex species according to the reported binary and ternary phase diagrams in the temperature range from 1773 to 1973 K (1500 to 1700 °C)^23^. The chemical reaction formulas of complex molecules that are possibly formed, their standard Gibbs free energy changes ${{\rm{\Delta }}}_{{\rm{r}}}{G}_{{\rm{m}},{\rm{c}}i}^{{\rm{\theta }}}$, the mole number *n*_*i*_, mass action concentration *N*_*i*_ and the equilibrium constant ${K}_{{\rm{c}}i}^{{\rm{\theta }}}$ in 100 g CaF_2_-CaO-Al_2_O_3_-MgO-TiO_2_ slags are listed in Table 1. The mass action concentration of complex molecules can be expressed by ${N}_{{{\rm{CaF}}}_{2}}$(*N*_1_), *N*_CaO_(*N*_2_), ${N}_{{{\rm{Al}}}_{2}{{\rm{O}}}_{3}}$(*N*_3_), *N*_MgO_(*N*_4_), ${N}_{{{\rm{TiO}}}_{2}}$(*N*_5_) and ${K}_{{\rm{c}}i}^{{\rm{\theta }}}$. (II) The activity coefficient *f*_%,*i*_ of Al and Ti can be calculated using the Wagner equation^18^; the interaction coefficients used in the present study are listed in Table 2. The chemical composition of Inconel 718 alloy is listed in Table 3. (III) The temperature range investigated during the ESR process was 1773 to 1973 K (1500 to 1700 °C).

**Table 1:** Chemical reaction formulas for possibly formed complex compounds, their standard Gibbs free energy changes, mole number, mass action concentration and equilibrium constants in 100 g CaO-MgO-Al_2_O_3_-TiO_2_-CaF_2_ slags.

Reactions	${{\boldsymbol{\Delta }}}_{{\bf{r}}}{{\boldsymbol{G}}}_{{\bf{m}},{\boldsymbol{c}}{\boldsymbol{i}}}^{{\boldsymbol{\theta }}}$ (J/mol)	Mole Number of Structural Units (mol)	Mass Action Concentration of Structural Units (−)	${{\boldsymbol{K}}}_{{\bf{c}}{\boldsymbol{i}}}^{{\boldsymbol{\theta }}}$ (−)
3(Ca^2+^ + O^2−^) + (Al_2_O_3_) = (3CaO·Al_2_O_3_)	−21,757–29.288 *T*	${n}_{{\rm{c}}1}={n}_{3{\rm{CaO}}\cdot {{\rm{Al}}}_{2}{{\rm{O}}}_{3}}$	${N}_{{\rm{c}}1}=\frac{{n}_{{\rm{c}}1}}{{\sum }^{}{n}_{i}}={N}_{3{\rm{CaO}}\cdot {{\rm{Al}}}_{2}{{\rm{O}}}_{3}}$	${K}_{{\rm{c}}1}^{{\rm{\theta }}}=\frac{{N}_{c1}}{{N}_{{\rm{CaO}}}^{3}{N}_{{{\rm{Al}}}_{2}{{\rm{O}}}_{3}}}$
12(Ca^2+^ + O^2−^) + 7(Al_2_O_3_) = (12CaO·7Al_2_O_3_)	617,977–612.119 *T*	${n}_{{\rm{c}}2}={n}_{12{\rm{CaO}}\cdot 7{{\rm{Al}}}_{2}{{\rm{O}}}_{3}}$	${N}_{{\rm{c}}2}=\frac{{n}_{{\rm{c}}2}}{{\sum }^{}{n}_{i}}={N}_{12{\rm{CaO}}\cdot 7{{\rm{Al}}}_{2}{{\rm{O}}}_{3}}$	${K}_{{\rm{c}}2}^{{\rm{\theta }}}=\frac{{N}_{{\rm{c}}2}}{{N}_{{\rm{CaO}}}^{12}{N}_{{{\rm{Al}}}_{2}{{\rm{O}}}_{3}}^{7}}$
(Ca^2+^ + O^2−^) + (Al_2_O_3_) = (CaO·Al_2_O_3_)	59,413–59.413 *T*	${n}_{{\rm{c}}3}={n}_{{\rm{CaO}}\cdot {{\rm{Al}}}_{2}{{\rm{O}}}_{3}}$	${N}_{{\rm{c}}3}=\frac{{n}_{{\rm{c}}3}}{{\sum }^{}{n}_{i}}={N}_{{\rm{CaO}}\cdot {{\rm{Al}}}_{2}{{\rm{O}}}_{3}}$	${K}_{{\rm{c}}3}^{{\rm{\theta }}}=\frac{{N}_{{\rm{c}}3}}{{N}_{{\rm{CaO}}}{N}_{{{\rm{Al}}}_{2}{{\rm{O}}}_{3}}}$
(Ca^2+^ + O^2−^) + 2(Al_2_O_3_) = (CaO·2Al_2_O_3_)	−16,736–25.522 *T*	${n}_{{\rm{c}}4}={n}_{{\rm{CaO}}\cdot 2{{\rm{Al}}}_{2}{{\rm{O}}}_{3}}$	${N}_{{\rm{c}}4}=\frac{{n}_{{\rm{c}}4}}{{\sum }^{}{n}_{i}}={N}_{{\rm{CaO}}\cdot 2{{\rm{Al}}}_{2}{{\rm{O}}}_{3}}$	${K}_{{\rm{c}}4}^{{\rm{\theta }}}=\frac{{N}_{{\rm{c}}4}}{{N}_{{\rm{CaO}}}{N}_{{{\rm{Al}}}_{2}{{\rm{O}}}_{3}}^{2}}$
(Ca^2+^ + O^2−^) + 6(Al_2_O_3_) = (CaO·6Al_2_O_3_)	−22,594–31.798 *T*	${n}_{{\rm{c}}5}={n}_{{\rm{CaO}}\cdot 6{{\rm{Al}}}_{2}{{\rm{O}}}_{3}}$	${N}_{{\rm{c}}5}=\frac{{n}_{{\rm{c}}5}}{{\sum }^{}{n}_{i}}={N}_{{\rm{CaO}}\cdot 6{{\rm{Al}}}_{2}{{\rm{O}}}_{3}}$	${K}_{{\rm{c}}5}^{{\rm{\theta }}}=\frac{{N}_{{\rm{c}}5}}{{N}_{{\rm{CaO}}}{N}_{{{\rm{Al}}}_{2}{{\rm{O}}}_{3}}^{4}}$
(Ca^2+^ + O^2−^) + (TiO_2_) = (CaO·TiO_2_)	−79,967.9–3.35 *T*	${n}_{{\rm{c}}6}={n}_{{\rm{CaO}}\cdot {{\rm{TiO}}}_{2}}$	${N}_{{\rm{c}}6}=\frac{{n}_{{\rm{c}}6}}{{\sum }^{}{n}_{i}}={N}_{{\rm{CaO}}\cdot {{\rm{TiO}}}_{2}}$	${K}_{{\rm{c}}6}^{{\rm{\theta }}}=\frac{{N}_{{\rm{c}}6}}{{N}_{{\rm{CaO}}}{N}_{{{\rm{TiO}}}_{2}}}$
3(Ca^2+^ + O^2−^) + 2(TiO_2_) = (3CaO·2TiO_2_)	−207,247–11.51 *T*	${n}_{{\rm{c}}7}={n}_{3{\rm{CaO}}\cdot 2{{\rm{TiO}}}_{2}}$	${N}_{{\rm{c}}7}=\frac{{n}_{{\rm{c}}7}}{{\sum }^{}{n}_{i}}={N}_{3{\rm{CaO}}\cdot 2{{\rm{TiO}}}_{2}}$	${K}_{{\rm{c}}7}^{{\rm{\theta }}}=\frac{{N}_{{\rm{c}}7}}{{N}_{{\rm{CaO}}}^{3}{N}_{{{\rm{TiO}}}_{2}}^{2}}$
4(Ca^2+^ + O^2−^) + 3(TiO_2_) = (4CaO·3TiO_2_)	−293,076–17.58 *T*	${n}_{{\rm{c}}8}={n}_{4{\rm{CaO}}\cdot 3{{\rm{TiO}}}_{2}}$	${N}_{{\rm{c}}8}=\frac{{n}_{{\rm{c}}8}}{{\sum }^{}{n}_{i}}={N}_{4{\rm{CaO}}\cdot 3{{\rm{TiO}}}_{2}}$	${K}_{{\rm{c}}8}^{{\rm{\theta }}}=\frac{{N}_{{\rm{c}}8}}{{N}_{{\rm{CaO}}}^{4}{N}_{{{\rm{TiO}}}_{2}}^{3}}$
(Mg^2+^ + O^2−^) + (Al_2_O_3_) = (MgO·Al_2_O_3_)	−18,828–6.276 *T*	${n}_{{\rm{c}}9}={n}_{{\rm{MgO}}\cdot {{\rm{Al}}}_{2}{{\rm{O}}}_{3}}$	${N}_{{\rm{c}}9}=\frac{{n}_{{\rm{c}}9}}{{\sum }^{}{n}_{i}}={N}_{{\rm{MgO}}\cdot {{\rm{Al}}}_{2}{{\rm{O}}}_{3}}$	${K}_{{\rm{c}}9}^{{\rm{\theta }}}=\frac{{N}_{{\rm{c}}9}}{{N}_{{\rm{MgO}}}{N}_{{{\rm{Al}}}_{2}{{\rm{O}}}_{3}}}$
(Mg^2+^ + O^2−^) + (TiO_2_) = (MgO·TiO_2_)	−26,376.8 + 3.14 *T*	${n}_{{\rm{c}}10}={n}_{{\rm{MgO}}\cdot {{\rm{TiO}}}_{2}}$	${N}_{{\rm{c}}10}=\frac{{n}_{{\rm{c}}10}}{{\sum }^{}{n}_{i}}={N}_{{\rm{MgO}}\cdot {{\rm{TiO}}}_{2}}$	${K}_{{\rm{c}}10}^{{\rm{\theta }}}=\frac{{N}_{{\rm{c}}10}}{{N}_{{\rm{MgO}}}{N}_{{{\rm{TiO}}}_{2}}}$
(Mg^2+^ + O^2−^) + 2(TiO_2_) = (MgO·2TiO_2_)	−27,632.9 + 0.63 *T*	${n}_{{\rm{c}}11}={n}_{{\rm{MgO}}\cdot 2{{\rm{TiO}}}_{2}}$	${N}_{{\rm{c}}11}=\frac{{n}_{{\rm{c}}11}}{{\sum }^{}{n}_{i}}={N}_{{\rm{MgO}}\cdot 2{{\rm{TiO}}}_{2}}$	${K}_{{\rm{c}}11}^{{\rm{\theta }}}=\frac{{N}_{{\rm{c}}11}}{{N}_{{\rm{MgO}}}{N}_{{{\rm{TiO}}}_{2}}^{2}}$
2(Mg^2+^ + O^2−^) + (TiO_2_) = (2MgO·TiO_2_)	−25,539.5 + 1.26 *T*	${n}_{{\rm{c}}12}={n}_{2{\rm{MgO}}\cdot {{\rm{TiO}}}_{2}}$	${N}_{{\rm{c}}12}=\frac{{n}_{{\rm{c}}12}}{{\sum }^{}{n}_{i}}={N}_{2{\rm{MgO}}\cdot {{\rm{TiO}}}_{2}}$	${K}_{{\rm{c}}12}^{{\rm{\theta }}}=\frac{{N}_{{\rm{c}}12}}{{N}_{{\rm{MgO}}}^{2}{N}_{{{\rm{TiO}}}_{2}}}$
(Al_2_O_3_) + (TiO_2_) = (Al_2_O_3_·TiO_2_)	−25,270 + 3.924 *T*	${n}_{{\rm{c}}13}={n}_{{{\rm{Al}}}_{2}{{\rm{O}}}_{3}\cdot {{\rm{TiO}}}_{2}}$	${N}_{{\rm{c}}13}=\frac{{n}_{{\rm{c}}13}}{{\sum }^{}{n}_{i}}={N}_{{{\rm{Al}}}_{2}{{\rm{O}}}_{3}\cdot {{\rm{TiO}}}_{2}}$	${K}_{{\rm{c}}13}^{{\rm{\theta }}}=\frac{{N}_{{\rm{c}}13}}{{N}_{{{\rm{Al}}}_{2}{{\rm{O}}}_{3}}{N}_{{{\rm{TiO}}}_{2}}}$
3(Ca^2+^ + O^2−^) + 3(Al_2_O_3_) + (Ca^2+^ + 2F^2−^) = (3CaO·2Al_2_O_3_·CaF_2_)	−44,492–73.15 *T*	${n}_{{\rm{c}}14}={n}_{3{\rm{CaO}}\cdot {{\rm{Al}}}_{2}{{\rm{O}}}_{3}\cdot {{\rm{CaF}}}_{2}}$	${N}_{{\rm{c}}14}=\frac{{n}_{{\rm{c}}14}}{{\sum }^{}{n}_{i}}={N}_{3{\rm{CaO}}\cdot {{\rm{Al}}}_{2}{{\rm{O}}}_{3}\cdot {{\rm{CaF}}}_{2}}$	${K}_{{\rm{c}}14}^{{\rm{\theta }}}=\frac{{N}_{{\rm{c}}14}}{{N}_{{\rm{CaO}}}^{3}{N}_{{{\rm{Al}}}_{2}{{\rm{O}}}_{3}}{N}_{{{\rm{CaF}}}_{2}}}$
11(Ca^2+^ + O^2−^) + 7(Al_2_O_3_) + (Ca^2+^ + 2F^2−^) = (11CaO·7Al_2_O_3_·CaF_2_)	−228,760–155.8 *T*	${n}_{{\rm{c}}15}={n}_{11{\rm{CaO}}\cdot 7{{\rm{Al}}}_{2}{{\rm{O}}}_{3}\cdot {{\rm{CaF}}}_{2}}$	${N}_{{\rm{c}}15}=\frac{{n}_{{\rm{c}}15}}{{\sum }^{}{n}_{i}}={N}_{11{\rm{CaO}}\cdot 7{{\rm{Al}}}_{2}{{\rm{O}}}_{3}\cdot {{\rm{CaF}}}_{2}}$	${K}_{{\rm{c}}15}^{{\rm{\theta }}}=\frac{{N}_{{\rm{c}}15}}{{N}_{{\rm{CaO}}}^{11}{N}_{{{\rm{Al}}}_{2}{{\rm{O}}}_{3}}^{2}{N}_{{{\rm{CaF}}}_{2}}}$

**Table 2:** Interaction coefficients used in the present study^32–35^.

${{\boldsymbol{e}}}_{{\boldsymbol{i}}}^{{\boldsymbol{j}}}$	Mn	Cr	Ni	Al	Ti	Mo
Al	0.034	0.045	−0.0376	0.08	—	—
Ti	−0.12	0.025	−0.0166	—	0.0561	0.016

**Table 3:** Composition of the Inconel 718 alloy used in the present study (wt%).

C	Si	Mn	Mo	Ni	Cr	Nb	Al	Ti	Fe
0.02	0.2	0.09	3.13	52.72	18.26	4.92	0.43	1.13	bal.

**Figure 2:**
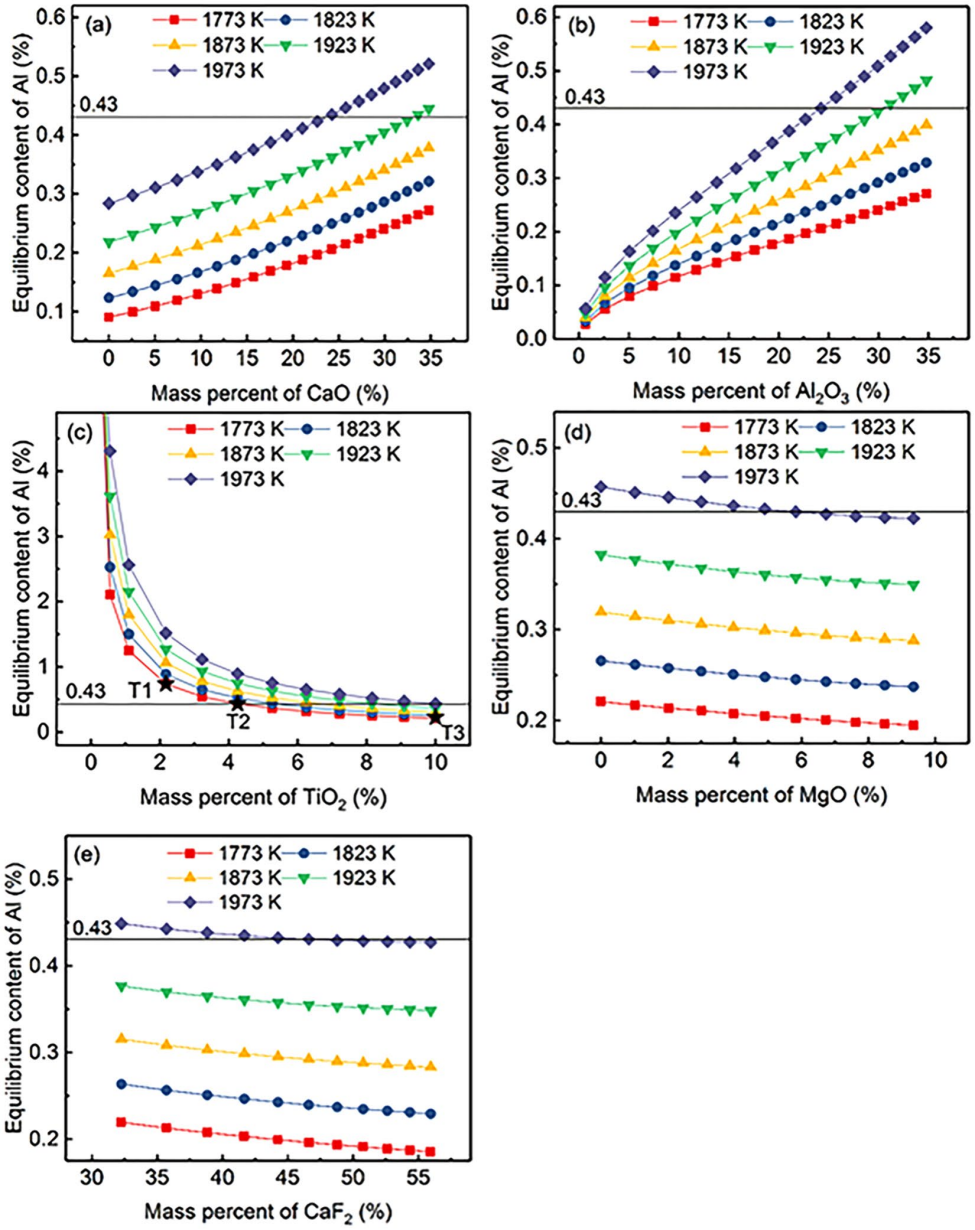
Relationship between equilibrium content of Al for a given Ti content (1.13) and the chemical composition of each component in CaF_2_-CaO-Al_2_O_3_-MgO-TiO_2_ slag at various temperatures.

The relationship between the calculated equilibrium Al content for a given Ti content (1.13) and the slag composition of component *i* in CaF_2_-CaO-Al_2_O_3_-MgO-TiO_2_ slags in the temperature range from 1773 to 1973 K (1500 to 1700 °C) is illustrated in Fig. 2. The equilibrium Al content increases with increasing temperature at a constant slag composition. The dependence of the determined equilibrium Al content on the CaO content in the slag (CaF_2_:*X*_CaO_:Al_2_O_3_:MgO:TiO_2_ = 37:*X*_CaO_:25:3:10) at different temperatures is illustrated in Fig. 2(a). The figure shows a continuous increase in the equilibrium Al content with increasing addition of CaO. The calculated equilibrium Al content is greater than 0.43 (the Al content in the master alloy used in the present study) under appropriate conditions, viz., the temperature is greater than 1923 K (1650 °C) and (% CaO) > 22.68, which indicates that Al is not subject to oxidation. This phenomenon is consistent with the results of Jiang *et al*.^24^, who reported that the high CaO content in CaF_2_-CaO-Al_2_O_3_-MgO-TiO_2_-SiO_2_ slags can prevent the oxidation of Al in GH8825 during the ESR process. Similar results are shown in Fig. 2(b). In Fig. 2(c), the calculated equilibrium Al content in the molten metal at various temperatures is plotted against the TiO_2_ content. The equilibrium Al content decreases sharply under conditions of (% TiO_2_) < 1.1, and the point of intersection of the equilibrium Al content line and the isoconcentration line ([% Al] = 0.43) is shifted to the high-TiO_2_-content side when the temperature is increased. Similarly, Hou *et al*.^25^ found that the slag containing a low CaO content combined with extra TiO_2_ constantly added into the molten slag in the first temperature-rising period is suitable for electroslag remelting of 1Cr21Ni5Ti stainless steel. Figure 2(d) illustrates the evolution of the calculated equilibrium Al content in the Ni-based alloy when the proportion of MgO in the molten slag is different (0 ≤ (% MgO) ≤ 9.35), which shows that the equilibrium Al content decreases with increasing MgO content. At the temperature of 1923 K (1650 °C), as Fig. 2(d) shows, the equilibrium Al content is always less than 0.43 over the full composition range of MgO, indicating that satisfying the smelting requirements of Inconel 718 alloy only by adjusting the mass content of MgO in the molten slag is difficult. However, the oxidation of Al in the Ni-based alloy can be damped when the mass percent of MgO is less than 5.83% at 1973 K (1700 °C). A similar behaviour in the dependence of the equilibrium Al content is observed in Fig. 2(e).

**Figure 3:**
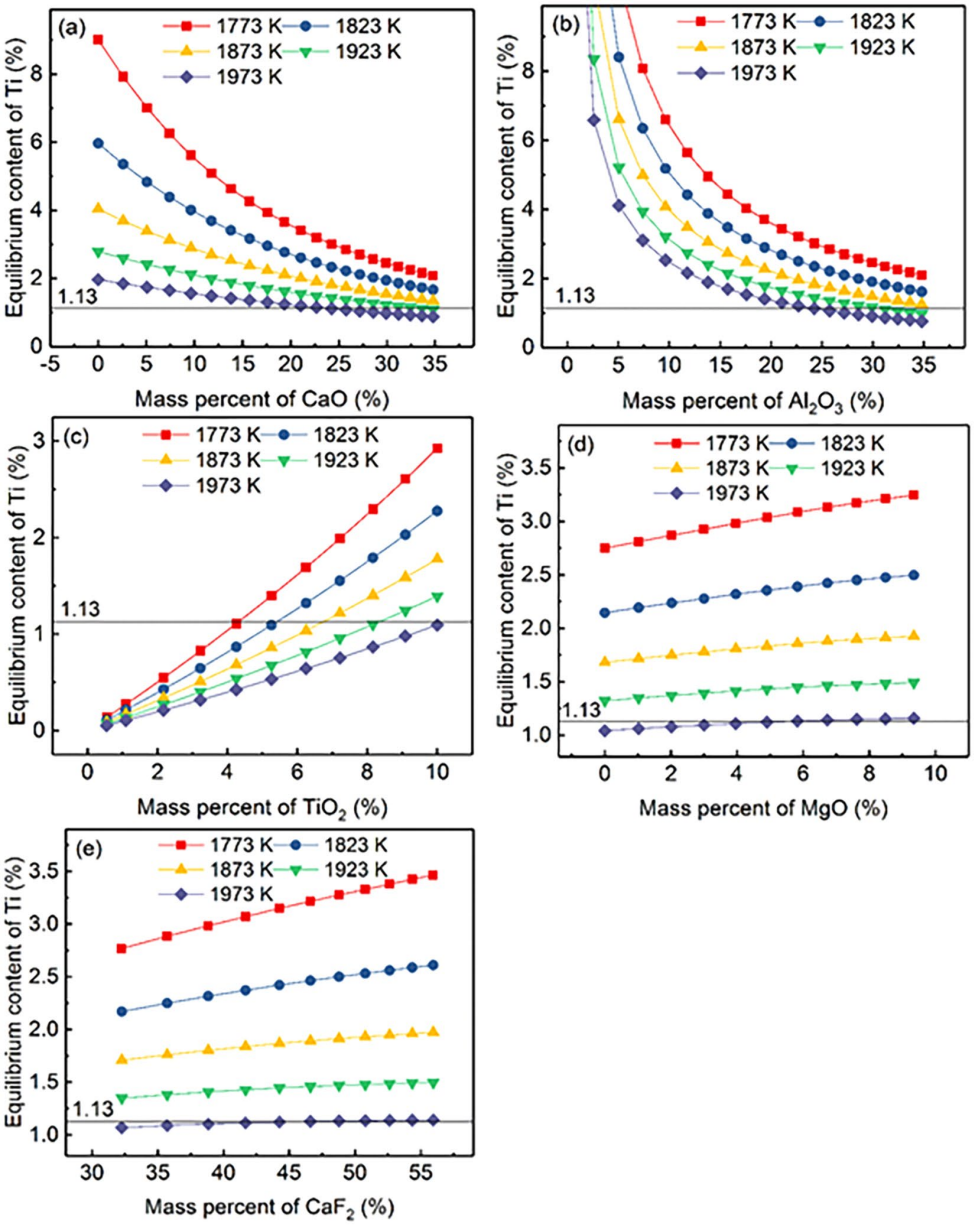
Relationship between the equilibrium content of Ti for a given Al content (0.43) and the chemical composition of each component in CaF_2_-CaO-Al_2_O_3_-MgO-TiO_2_ slag at various temperatures.

The relationship between calculated equilibrium Ti content for a given Al content (0.43) and the slag composition of component *i* in CaF_2_-CaO-Al_2_O_3_-MgO-TiO_2_ slags in the temperature range from 1773 to 1973 K (1500 to 1700 °C) is illustrated in Fig. 3. It can be observed that the equilibrium Ti content shows negative correlation with temperature in the range of 1773 to 1973 K (1500 to 1700 °C) with constant slag composition. Meanwhile, the effect of mass percent for CaO, Al_2_O_3_, TiO_2_, MgO, and CaF_2_ as components in CaF_2_-CaO-Al_2_O_3_-MgO-TiO_2_ slags on the equilibrium Ti content illustrated in Fig. 3 shows completely opposite trend compared with Fig. 2.

As it is previously discussed, the effects of MgO and CaF_2_ on controlling the equilibrium Al content in Ni-based alloy compared that of TiO_2_, CaO, and Al_2_O_3_ in CaF_2_-CaO-Al_2_O_3_-MgO-TiO_2_ slags are very mild. Zaitsev *et al*.^26,27^ also demonstrated that the activities of CaO and Al_2_O_3_ were not substantially changed by addition of CaF_2_ to the CaF_2_-CaO-Al_2_O_3_ slag system. However, CaF_2_ plays an important role in the ESR process to improve the fluidity and conductivity of the slag^28^. Therefore, the importance of factors for controlling the equilibrium Al content in Inconel 718 is ordered as TiO_2_ > Al_2_O_3_ > CaO > CaF_2_ > MgO in ESR slags.

**Figure 4:**
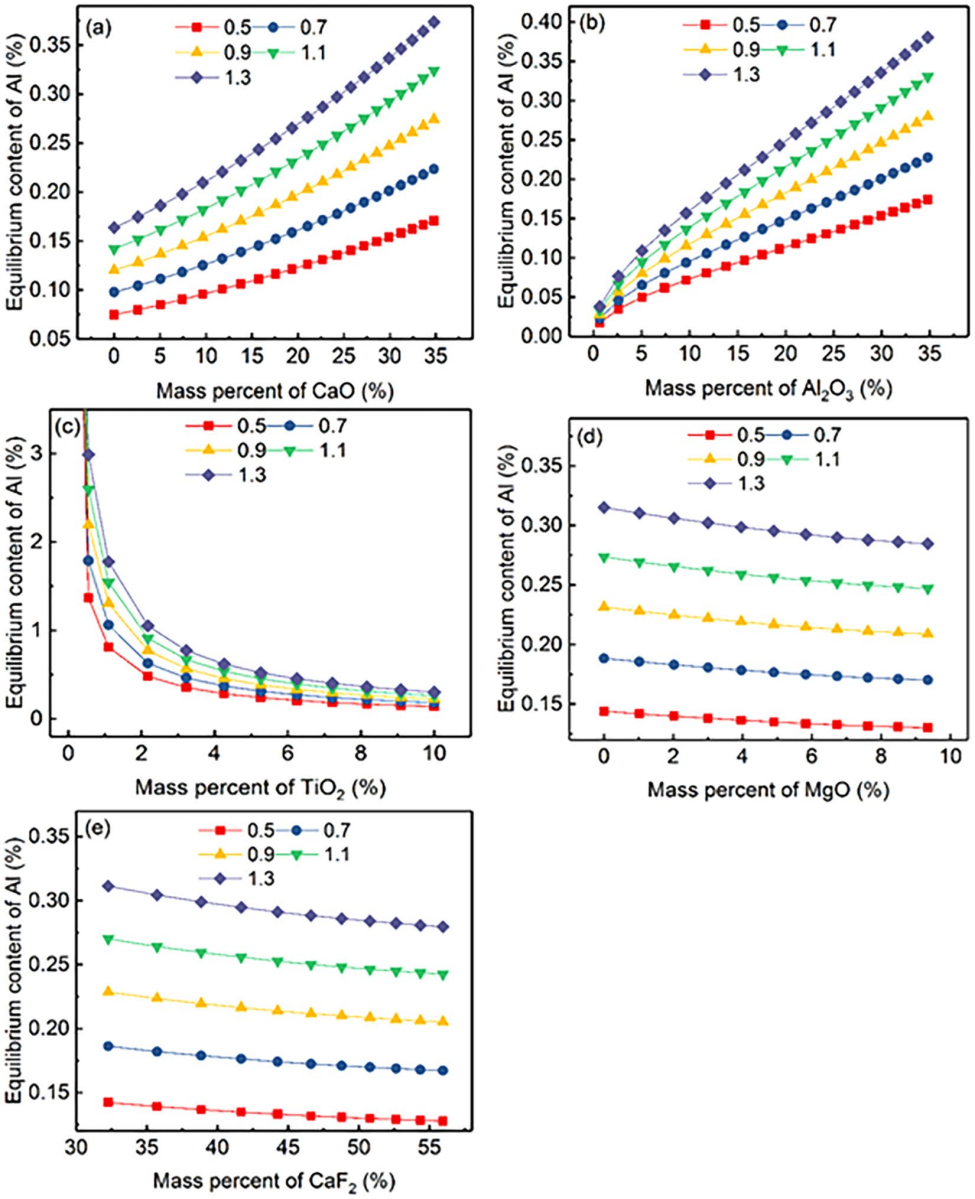
Relationship between equilibrium content of Al for various Ti contents and the chemical composition of each component in CaF_2_-CaO-Al_2_O_3_-MgO-TiO_2_ slag under various Ti content at 1873 K (1600 °C).

**Figure 5:**
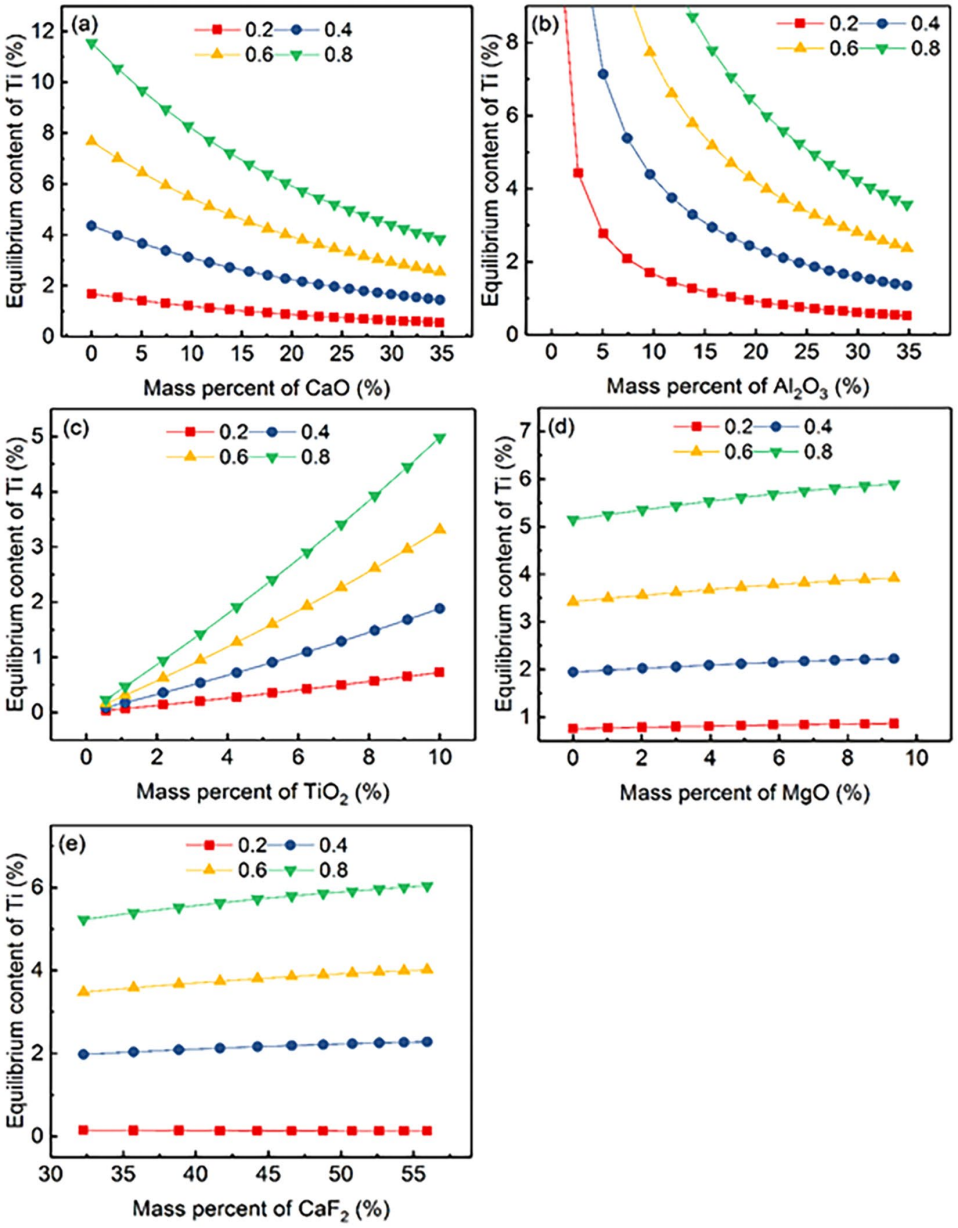
Relationship between the equilibrium content of Ti for various Al contents and the chemical composition of each component in CaF_2_-CaO-Al_2_O_3_-MgO-TiO_2_ slag under various Al contents at 1873 K (1600 °C).

To elaborate the Inconel 718 alloy composition design, the influence of slag composition on the calculated equilibrium Al content for five different Ti contents (0.5, 0.7, 0.9, 1.1 and 1.3) at 1873 K (1600 °C) is shown in Fig. 4, from which it can be found that all above mentioned observations in Fig. 2 are consistent with the trend shown in Fig. 4. The equilibrium Al content increases with increasing Ti content for a given slag composition at a constant temperature, which reveals that, on the prerequisite of satisfying the mechanical properties of the alloy, to properly increases Ti content can prevent oxidation of Al in the Inconel 718 superalloy. The influence of slag composition on the calculated equilibrium Ti content is shown in Fig. 5, which shows that the calculated equilibrium Ti content increases with increasing Al content (0.2, 0.4, 0.6 and 0.8). The determined equilibrium Ti content is greater than 1.13 for a fixed Al content at 0.8 in the full composition range of slag composition of component *i* in CaF_2_-CaO-Al_2_O_3_-MgO-TiO_2_ slags ((% TiO_2_) > 3.23). However, the determined Al content for the upper limit Ti content (1.3) is less than 0.43 over the full composition range of slag composition ((% TiO_2_) > 6.25) shown in Fig. 4. These results demonstrate that the Al and Ti content in the consumable electrode can be designed to be the upper limit in the case where some mechanical properties are met, which is an effective method of controlling Al and Ti loss during the ESR process. What’s more, it can be obtained by comparing Figs 4 and 5 that the importance of factors for preventing oxidation of active elements in Inconel 718 is ordered as Al > Ti in the consumable electrode.

## Experimental Materials and Methods

### Metal and slag preparation

To begin the experiment, Inconel 718 alloy samples were produced in a MgO crucible in a vacuum-induction melting (VIM) furnace under a high-purity argon atmosphere; the chemical composition is listed in Table 3. Each of the master alloys was cut into a smaller size (1 to 3 cm^3^) to facilitate weighing. Analar-grade CaF_2_, CaO, MgO, Al_2_O_3_, and TiO_2_ were used as raw materials. The thoroughly mixed powders were melted at 1773 K (1500 °C) in a graphite crucible under a high-purity Ar atmosphere to ensure complete melting and homogenization; the liquid sample was then quenched on the cooled copper plate and ground. The chemical composition of the experimental slag is listed in Table 4.

**Table 4:** Composition of slag used in this study and measured equilibrium content of Ni-based alloy samples at each experimental heat (wt%).

Run No.	CaF_2_	CaO	Al_2_O_3_	MgO	TiO_2_	Measured Equilibrium Content	
						Al	Ti
T1	40.22	27.17	27.17	3.26	2.17	0.65	0.95
T2	39.36	26.6	26.6	3.16	4.26	0.41	1.15
T3	37	25	25	3	10	0.23	1.50

### Experimental apparatus and process

**Figure 6:**
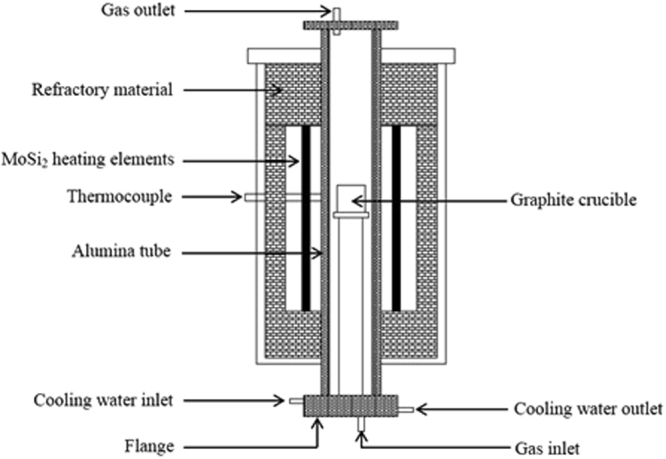
Schematic of the experimental apparatus used in the present study.

**Figure 7:**
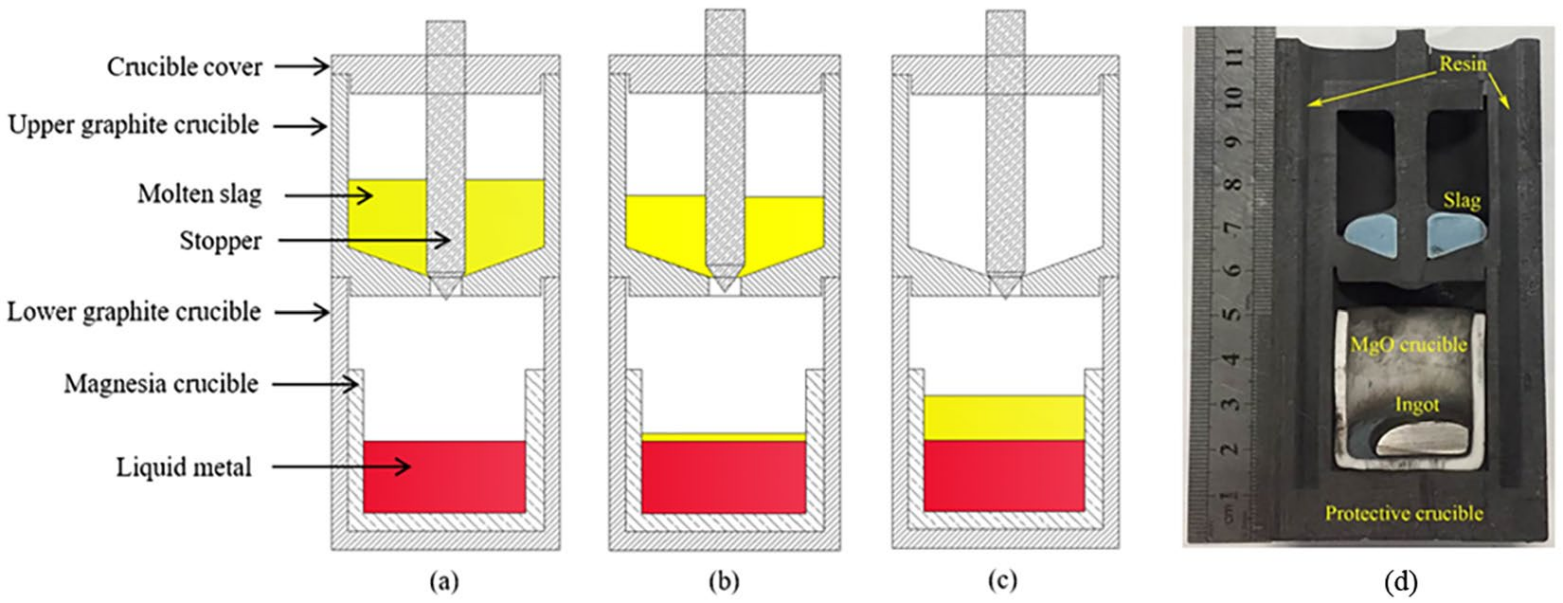
Schematic of double-layer crucible: (**a**)melting process, (**b**) experiment starts, (**c**) slag-metal reaction and (**d**) equipment image.

**Figure 8:**
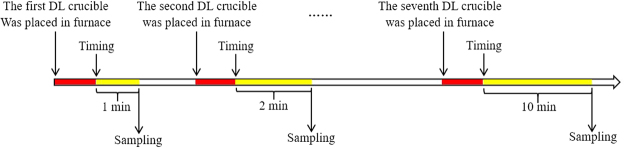
Flow sheet of the experimental procedure.

The slag-metal equilibrium reaction between CaF_2_-CaO-Al_2_O_3_-MgO-TiO_2_ slags and Inconel 718 alloy was conducted in a vertical resistance-heated alumina tube furnace equipped with MoSi_2_ heating elements. Figure 6 shows a schematic of the resistance furnace used in the present study. The temperature of the furnace was controlled by a proportional-integral-derivative (PID) controller connected to a B-type reference thermocouple. The temperature was calibrated to 1773 K (1500 °C) using another B-type thermocouple before the experiment. The kinetic experiments were carried out using the double-layer (DL) graphite crucible shown in Fig. 7. The experimental procedure is briefly summarized in steps as follows:

1. Pre-melted slag (12 g) was held in the upper graphite crucible with a small hole in its bottom after a carbon stopper was used to plug the hole. The inner wall of the upper crucible makes an angle of 110 degrees with the bottom to ensure that the melted slag drops fully into the lower separate crucible. Face-cleaned Inconel 718 alloy (22 g) was accommodated in a MgO crucible (ID: 25 mm; OD: 30 mm; H: 35 mm). The MgO crucible was then placed in the lower graphite crucible (ID: 31 mm; OD: 36 mm; H: 40 mm) to prevent the reactant from leaking.
2. The DL crucible with reactants was placed in a graphite tray, which was then positioned in the constant-temperature zone of a resistance furnace after the furnace temperature reached the pre-set temperature of 1773 K (1500 °C). High-purity Ar was flushed into the reaction chamber at a constant flow rate to avoid the oxidation of the Ni-based alloy.
3. Prior to the experiment, the time required for the slag and metal to melt completely was estimated; these estimates indicated that the samples had fully melted after the graphite crucible was placed into the resistance furnace for 10 min.
4. The moment the carbon stopper was removed (viz., the moment contact occurred between the molten slag and metal) was taken as the starting time of the reaction. After certain reaction time intervals (1, 2, 3, 4, 5, 7 and 10 min), the whole crucible containing liquid samples was rapidly removed from the furnace and quenched in ice water. The flow sheet for the experimental procedure is shown in Fig. 8. After completion of the experiments, the composition of the Ni-based alloy samples was analysed by inductively coupled plasma atomic emission spectroscopy (ICP-AES).

## Results and Discussion

### Oxidation behaviour of Al and Ti through CaF_2_-CaO-Al_2_O_3_-MgO-TiO_2_ slag

**Figure 9:**
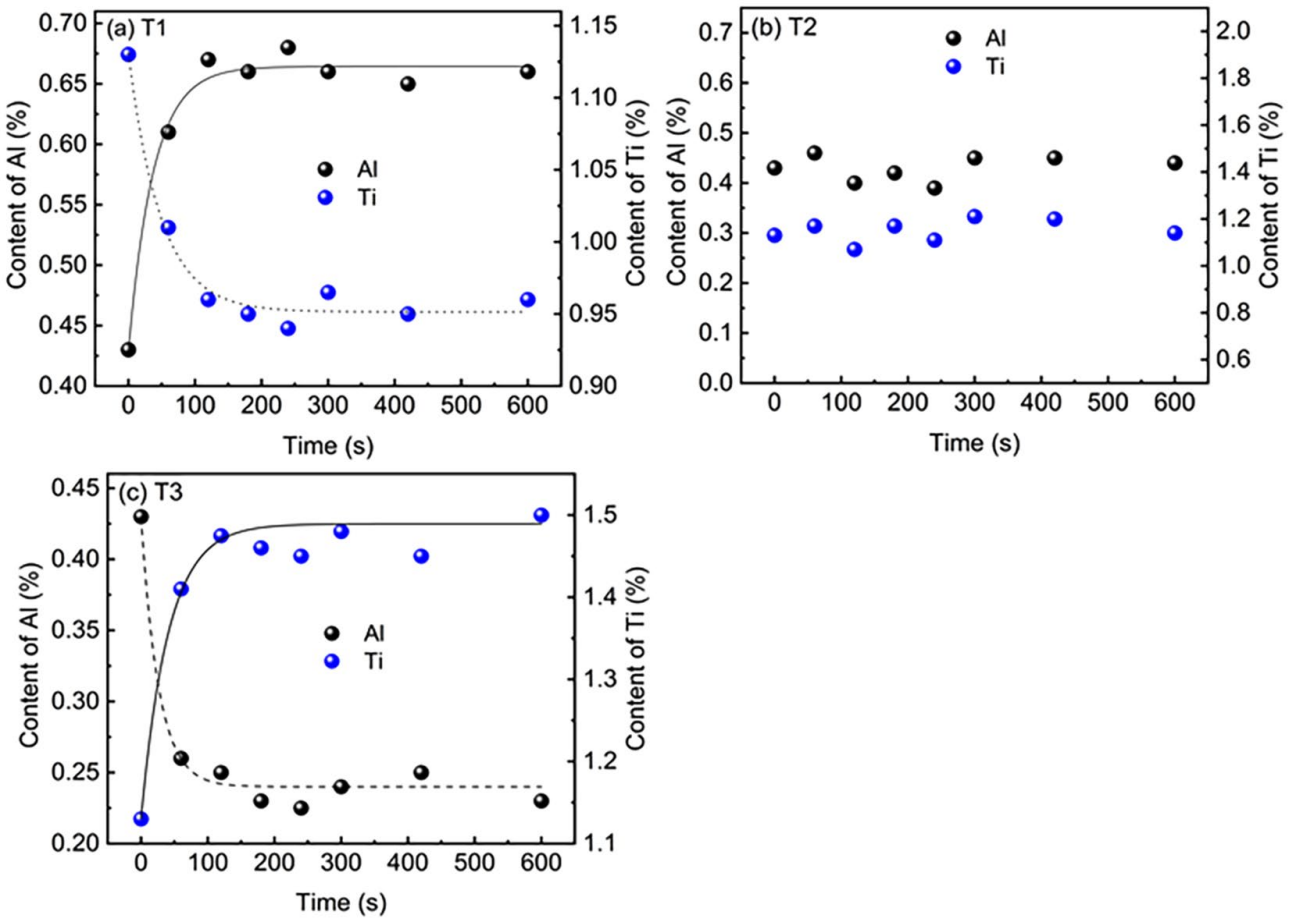
The Al and Ti content in the Ni-based alloy as a function of reaction time for various slags (**a**) T1, (**b**) T2 and (**c**) T3 at 1773 K (1500 °C).

According to the calculated results in the section on ‘Relationship between equilibrium content of Al or Ti and slag composition at different temperatures’, three laboratory-scale experiments (T1, T2 and T3) with different slag compositions (Fig. 2(c)) were performed at 1773 K (1500 °C). The changes in concentration of Al and Ti in the metal phase are shown in Fig. 9 as a function of time for various slags. Figure 7(a) shows that the Al_2_O_3_ in molten slag was rapidly reduced by Ti during the first 3–5 min, after which the Al_2_O_3_ remained nearly constant. Similar results are also observed in Fig. 7(c). The Al and Ti contents remained constant as functions of time. Full details of the experimental results are given in Table 4. These results indicate that the calculated equilibrium content of Al and Ti in the metal phase shows reliable agreement with the measured values.

### Establishment of the kinetic model

The interface chemical reaction is generally accepted to be rapid at metallurgical temperatures, which gives rise to the fact that the overall reaction rate is controlled only by mass transfer of a species to or from the slag-metal interface through the concentration boundary layer^17^. On the basis of this theory of Wagner^29^, the main assumptions in the developed kinetic model for elucidating the probable reaction mechanism between molten slag and Ni-based alloy can be briefly summarized as follows: (I) Only two phases (liquid slag and Ni-based alloy) are considered. (II) The interface chemical reaction reaches a local equilibrium at metallurgical temperatures, and this equilibrium does not control the overall rate of reaction. (III) Reactant and product do not accumulate at the slag-metal interface, and the mass transfer resistance occurs mainly at the boundary layer.

Therefore, reaction (1) may be divided into the following elementary steps:

1. Mass transfer of [Al] in the Ni-based alloy from the bulk to the slag-metal interface: [Al] → [Al]^*^;
2. Mass transfer of (TiO_2_) in the molten slag from the bulk to the slag-metal interface: (TiO_2_) → (TiO_2_)^*^;
3. Interface chemical reaction: 4[Al]^*^ + 3(TiO_2_)^*^ = 3[Ti]^*^ + (Al_2_O_3_)^*^;
4. Mass transfer of [Ti]^*^ in the Ni-based alloy from the slag-metal interface to the bulk: [Ti]^*^ → [Ti];
5. Mass transfer of (Al_2_O_3_)^*^ in the Ni-based alloy from the slag-metal interface to the bulk: (Al_2_O_3_)^*^ → (Al_2_O_3_).

**Figure 10:**
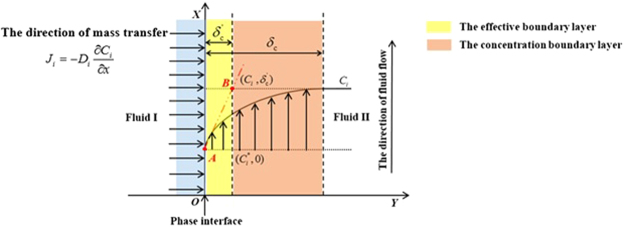
Schematic of the concentration ${C}_{i}^{{\rm{b}}}$ distribution during the heterogeneous reaction and definition of the thickness of the effective boundary layer ${\delta }_{{\rm{c}}}^{\text{'}}$ proposed by Wagner.

According to the fundamental equation of heterogeneous reaction kinetics based on the concept of the effective boundary layer, the flux of component *i* across unit area *J*_*i*_ is defined as Eq. (4). A schematic of the effective boundary layer is shown in Fig. 10. The derivation process of the fundamental equation of heterogeneous reaction kinetics has been presented in detail elsewhere^18^.

4 $${J}_{i}=\frac{{D}_{i}}{{\delta }_{c}^{\text{'}}}({C}_{i}^{\ast }-{C}_{i})={k}_{i}({C}_{i}^{\ast }-{C}_{i})\,({\rm{mol}}/{{\rm{m}}}^{2}\cdot {\rm{s}})$$

If the mass transfer of Ti in liquid metal is the rate-controlling step, the following equation can be deduced by inserting a relationship between the molar concentration and mass concentration terms:

5 $$\frac{{\rm{d}}[ \% \,{\rm{Ti}}]}{{\rm{dt}}}=\frac{{D}_{{\rm{Ti}}}}{{\delta }_{{\rm{Ti}}}{h}_{{\rm{m}}}}({[ \% {\rm{Ti}}]}^{\ast }-[ \% \,\,{\rm{Ti}}])$$

where *D*_Ti_, *δ*_Ti_ and *h*_m_ represent the diffusion coefficient of Ti (m^2^/s), the thickness of the boundary layer in the metal phase (m), and the thinness of the molten metal (m). The equilibrium constant of the interface chemical reaction (1) can be written as:

6 $$K=\frac{{a}_{ \% ,{\rm{Ti}}}^{3}{a}_{{\rm{R}},{{\rm{Al}}}_{2}{{\rm{O}}}_{3}}^{2}}{{a}_{ \% ,\,{\rm{Al}}}^{4}{a}_{{\rm{R}},{{\rm{TiO}}}_{2}}^{3}}=\frac{{f}_{ \% ,{\rm{Ti}}}^{3}{[ \% {\rm{Ti}}]}^{\ast 3}{a}_{{\rm{R}},{{\rm{Al}}}_{2}{{\rm{O}}}_{3}}^{2}}{{f}_{ \% ,\,{\rm{Al}}}^{4}{\gamma }_{{{\rm{TiO}}}_{2}}^{3}{[ \% {\rm{Al}}]}^{\ast 4}{x}_{{{\rm{TiO}}}_{2}}^{\ast 3}}$$

where *a*_%,*i*_ and *f*_%,*i*_ are the activity and activity coefficient of composition *i* in a metal referred to the 1% standard state, with mass percentage [% *i*] as the concentration unit (−) and *a*_R,*i*_ and *γ*_*i*_ are the activity and activity coefficient of composition *i* in the slag relative to pure matter as a standard state, with mole fraction *x*_*i*_ as the concentration unit (−).

In view of the small change in the composition of the components included in reaction (1) (*e*.*g*., Al, Ti, Al_2_O_3_, and TiO_2_), the activity coefficient for each aforementioned component can be regarded as approximatively constant. In particular, high concentrations of Al_2_O_3_ are found in the present study, indicating that the activity ${a}_{{{\rm{Al}}}_{2}{{\rm{O}}}_{3}}$ of Al_2_O_3_ can also be treated as a constant. These constants are hence incorporated into the equilibrium constant *K* of reaction (1). The collated equilibrium constant *K*_1_ can be expressed as

7 $${K}_{1}=\frac{{[ \% {\rm{Ti}}]}^{\ast 3}}{{[ \% {\rm{Al}}]}^{\ast 4}{( \% {{\rm{TiO}}}_{2})}^{\ast 3}}$$

8 $${[ \% {\rm{Ti}}]}^{\ast 3}={( \% {{\rm{TiO}}}_{2})}^{\ast }\sqrt[3]{{K}_{1}{[ \% {\rm{Al}}]}^{\ast 4}}$$

The maximum mass transfer rate of Ti in liquid metal can be obtained by substituting the mass percent at the slag-metal interface, *i*.*e*. [% Al]^*^ and (% TiO_2_)^*^, with the mass concentration in bulk, as shown in Eq. (9):

9 $$\frac{{\rm{d}}[ \% \,{\rm{Ti}}]}{{\rm{d}}t}=\frac{{D}_{{\rm{Ti}}}}{{\delta }_{{\rm{Ti}}}{h}_{{\rm{m}}}}(( \% \,{{\rm{TiO}}}_{2})\sqrt[3]{{K}_{1}{[ \% {\rm{Al}}]}^{4}}-[ \% \,{\rm{Ti}}])$$

The concentration [% Al] of Al in the bulk metal and the (% TiO_2_) of TiO_2_ in the bulk slag in Eq. (9) can be associated with the [% Ti] in the bulk metal via a stoichiometric relationship at any time during the reaction process:

10 $$( \% \,{{\rm{TiO}}}_{2})={( \% {{\rm{TiO}}}_{2})}^{0}-\frac{{W}_{{\rm{m}}}}{{W}_{{\rm{s}}}}\frac{{M}_{{{\rm{TiO}}}_{2}}}{{M}_{{\rm{Ti}}}}[ \% \,{\rm{Ti}}]={( \% {{\rm{TiO}}}_{2})}^{0}-{Q}_{1}[ \% \,{\rm{Ti}}]$$

11 $$[ \% \,{\rm{Al}}]={[ \% {\rm{Al}}]}^{0}-\frac{4}{3}\frac{{M}_{{\rm{Al}}}}{{M}_{{\rm{Ti}}}}[ \% \,{\rm{Ti}}]={[ \% {\rm{Al}}]}^{0}-0.75\,[ \% \,{\rm{Ti}}]$$

where (% TiO_2_)^0^ and [% Al]^0^ represent the total amounts of TiO_2_ in the slag and Al in the molten metal, respectively. Combining Eq. (9) with Eqs (10) and (11), we obtain the following equation after simple mathematical derivation:

12 $${\int }_{0}^{{\rm{\Delta }}[{\rm{pct}}\,\text{Ti}]}\frac{{\rm{d}}[{\rm{pct}}\,{\rm{Ti}}]}{[{(\text{pct}{{\rm{TiO}}}_{2})}^{0}-{Q}_{1}({\rm{pct}}\,{\rm{Ti}})]\sqrt[3]{{K}_{1}{[{[\text{pct}\text{Al}]}^{0}-0.75[{\rm{pct}}{\rm{Ti}}]]}^{4}}-[{\rm{pct}}\,{\rm{Ti}}]}=\frac{{D}_{{\rm{Ti}}}}{{\delta }_{{\rm{Ti}}}{h}_{{\rm{m}}}}t$$

where Δ[% Ti] = [% *Ti*]_*t* = *t*_ − [% *Ti*]_*t* = 0_ is the difference between the concentration of Ti in the liquid metal at time *t* and that at time zero. The model for TiO_2_ diffusion in the slag, Al diffusion in the metal, and Al_2_O_3_ diffusion through a slag boundary layer have also been derived with respect to the Ti content in the bulk metal in a parallel manner:

13 $${\int }_{0}^{{\rm{\Delta }}[ \% \,{\rm{Ti}}]}\frac{{Q}_{1}d[ \% \,{\rm{Ti}}]}{[{( \% {{\rm{TiO}}}_{2})}^{0}-{Q}_{1}[ \% \,{\rm{Ti}}]]-\frac{[ \% \,{\rm{Ti}}]}{\sqrt[3]{{K}_{1}{[{[ \% {\rm{Al}}]}^{0}-0.75[ \% {\rm{Ti}}]]}^{4}}}}=\frac{{D}_{{{\rm{TiO}}}_{2}}}{{\delta }_{{{\rm{TiO}}}_{2}}{h}_{{\rm{s}}}}t$$

For mass transfer of Al in metal phase control,

14 $${\int }_{0}^{{\rm{\Delta }}[ \% \,{\rm{Ti}}]}\frac{0.75d[ \% \,\,{\rm{Ti}}]}{[{[ \% {\rm{Al}}]}^{0}-0.75[ \% \,{\rm{Ti}}]]-\sqrt[4]{\frac{{[ \% {\rm{Ti}}]}^{3}}{{K}_{1}{[{( \% {{\rm{TiO}}}_{2})}^{0}-{Q}_{1}[ \% {\rm{Ti}}]]}^{3}}}}=\frac{{D}_{{\rm{Al}}}}{{\delta }_{{\rm{Al}}}{h}_{{\rm{m}}}}t$$

For mass transfer of Al_2_O_3_ in slag phase control,

15 $${\int }_{0}^{{\rm{\Delta }}[ \% \,{\rm{Ti}}]}\frac{{Q}_{2}d[ \% \,{\rm{Ti}}]}{{[{[ \% {\rm{Al}}]}^{0}-0.75[ \% {\rm{Ti}}]]}^{2}\sqrt{\frac{{K}_{2}{[{( \% {{\rm{TiO}}}_{2})}^{0}-{Q}_{1}[ \% {\rm{Ti}}]]}^{3}}{{[ \% {\rm{Ti}}]}^{3}}-[{({{\rm{Al}}}_{2}{{\rm{O}}}_{3})}^{0}+{Q}_{2}[ \% \,{\rm{Ti}}]]}}=\frac{{D}_{{{\rm{Al}}}_{2}{{\rm{O}}}_{3}}}{{\delta }_{{{\rm{Al}}}_{2}{{\rm{O}}}_{3}}{h}_{{\rm{s}}}}t$$

where *K*_2_ = ([% Ti]^*3^(% Al_2_O_3_)^*2^)/([% Al]^*4^(% TiO_2_)^*3^) and ${Q}_{2}=\frac{2}{3}\frac{{W}_{{\rm{m}}}}{{W}_{{\rm{s}}}}\frac{{M}_{{{\rm{Al}}}_{2}{{\rm{O}}}_{3}}}{{M}_{{\rm{Ti}}}}$.

Inspection of Eqs (12)–(15) shows that they can both be represented as a function of mass concentration of Ti in the bulk metal:

16 $$F[( \% \,{\rm{Ti}})]=kt$$

When the maximum mass transfer rate models for Ti and Al in the metal (Eqs (12) and (14)) and TiO_2_ and Al_2_O_3_ in the slag (Eqs (13) and (15)) are valid, the plot of *F*[(% Ti)] versus time *t* should give a straight line and the mass transfer coefficient *k*_*i*_ can be acquired from its slope. For this purpose, the regressed formula of [% Ti] in the metal phase against the reaction time *t* can be expressed as a nonlinear function:

17 $$[ \% \,{\rm{Ti}}]=-0.36\,\exp (-t/41.05)+1.49\,{R}^{2}=0.98$$

**Figure 11:**
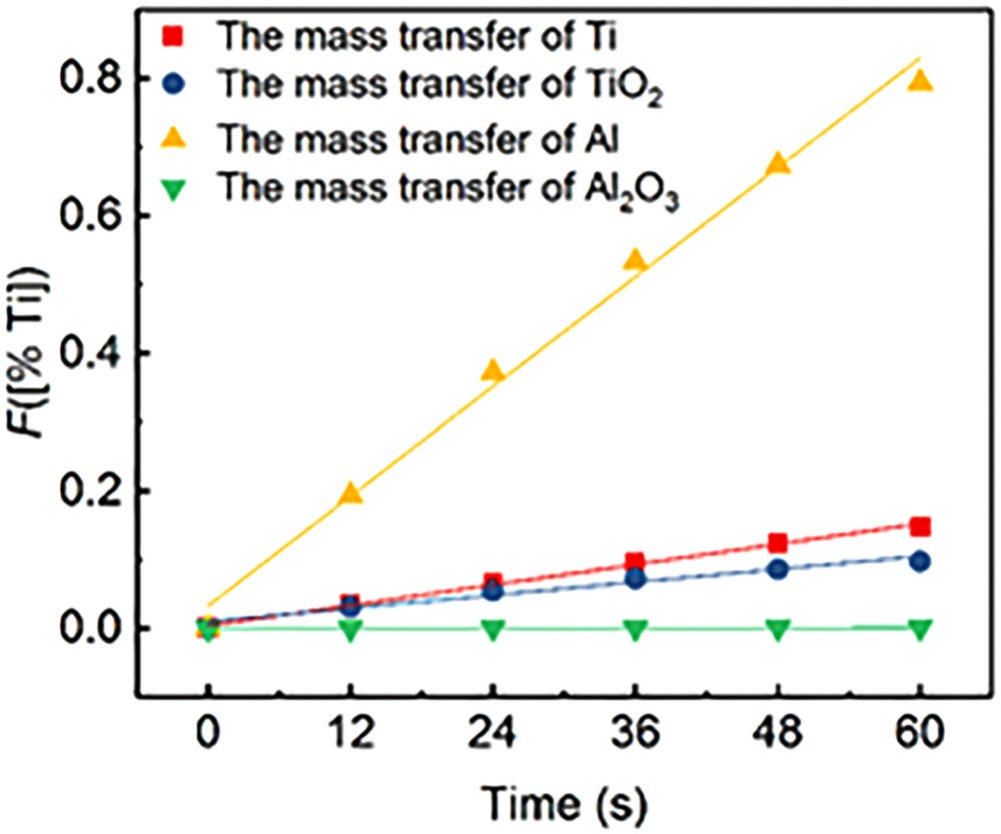
Determination of the mass transfer coefficient for S3 at 1773 K (1500 °C).

Figure 11 shows the data from Fig. 7(c) replotted according to Eqs (12)–(15). Although the experimental data appears to fit the line well, only the linear relationship between *F*[(%Ti)] and time *t* does not assure the mass transfer in the metal phase or the slag phase of being RCS. Therefore, the RCS and apparent activation energy of oxidation of Al and Ti in the Ni-based alloy by ESR slags will be carried out in the future. Certainly, the mass transfer rate of Al and Ti in the metal phase is much larger than the mass transfer rate of TiO_2_ and Al_2_O_3_ in the slag phase, rendering the mass transfer of TiO_2_ and/or Al_2_O_3_ in the slag phase likely to be the rate-controlling step. These results have been experimentally confirmed by several authors^25,30,31^. Recently, Hou *et al*.^25^ reported on the effect of slag composition on the oxidation kinetics of alloy elements during ESR of stainless steel experimentally using a 50-kg ESR furnace. They concluded that the rate-determining step of the oxidation reaction was the mass transfer of Al_2_O_3_ through the molten metal, SiO_2_ through the slag and TiO_2_ through both the metal and the slag phase.

## Conclusions

In the present study, the thermodynamic analysis for the slag-metal equilibrium reaction between Inconel 718 Ni-based alloy and CaF_2_-CaO-Al_2_O_3_-MgO-TiO_2_ ESR-type slags at various temperatures was systematically discussed. Meanwhile, the reaction mechanism was also investigated, coupling with the max mass transfer rate model. Our results are summarized as follows.

1. The equilibrium Al content showed a positive correlation with temperature in the range from 1773 to 1973 K (1500 to 1700 °C) with a constant slag composition, whereas the equilibrium Ti content showed the opposite trend.
2. The equilibrium Al and Ti contents depended weakly on the CaF_2_ and MgO contents in the studied slags, irrespective of the temperature, indicating that MgO can be used to tailor the physicochemical properties of the slags.
3. The thermodynamic results obtained from the IMCT calculation were in good agreement with the measurement results. The importance of factors for controlling the oxidation of Al and Ti in Inconel 718 superalloy was TiO_2_ > Al_2_O_3_ > CaO > CaF_2_ > MgO in ESR-type slag and Al > Ti in the consumable electrode.
4. The calculated results for controlling oxidation of reactive elements in the Ni-based alloy were in good agreement with the experimental results. An appropriate amount of TiO_2_ additive in the ESR slag can be quantitatively determined so that Al and Ti are not subject to oxidation during the ESR process.
5. The kinetic model was applied to the oxidation of Al in the Ni-based alloy by ESR slags. The results indicated that the mass transfer rate of Al and Ti in the metal phase was much larger than the mass transfer rate of TiO_2_ and Al_2_O_3_ in the slag phase.

### Data availability statement

All data generated or analysed during this study are included in this published article (and its Supplementary Information files).
